# Extracellular HCV-Core Protein Induces an Immature Regulatory Phenotype in NK Cells: Implications for Outcome of Acute Infection

**DOI:** 10.1371/journal.pone.0103219

**Published:** 2014-07-30

**Authors:** Lucy Golden-Mason, Young S. Hahn, Michael Strong, Linling Cheng, Hugo R. Rosen

**Affiliations:** 1 Division of Gastroenterology & Hepatology & Hepatitis C Center, University of Colorado, Aurora, Colorado, United States of America; 2 Beirne Carter Center for Immunology Research, Department of Microbiology & Pathology, University of Virginia, Charlottesville, Virginia, United States of America; 3 Bioinformatics, Department of Medicine, University of Colorado, Aurora, Colorado, United States of America; 4 Denver Veterans Affairs (VA), Denver, Colorado, United States of America; Saint Louis University, United States of America

## Abstract

**Background:**

Hepatitis C viral (HCV) proteins, including core, demonstrate immuno-modulatory properties; however, the effect of extracellular core on natural killer (NK) cells has not previously been investigated.

**Aims:**

To characterise NKs in acute HCV infection over time, and, to examine the effect of exogenous HCV-core protein on NK cell phenotype and function.

**Methods:**

Acute HCV patients (n = 22), including 10 subjects who spontaneously recovered, were prospectively studied. Flow-cytometry was used to measure natural cytotoxicity and to phenotype NKs directly *ex vivo* and after culture with HCV-core protein. Microarray analysis was used to identify pathways involved in the NK cell response to exogenous HCV-core.

**Results:**

Direct *ex vivo* analysis demonstrated an increased frequency of immature/regulatory CD56^bright^ NKs early in acute HCV infection *per se* which normalized with viral clearance. Natural cytotoxicity was reduced and did not recover after viral clearance. There was a statistically significant correlation between the frequency of CD56^bright^ NKs and circulating serum levels of HCV core protein. *In vitro* culture of purified CD56^bright^ NK cells with HCV-core protein in the presence of IL-15 maintained a significant proportion of NKs in the CD56^bright^ state. The *in vitro* effect of core closely correlates with NK characteristics measured directly *ex vivo* in acute HCV infection. Pathway analysis suggests that HCV-core protein attenuates NK interferon type I responses.

**Conclusions:**

Our data suggest that HCV-core protein alters NK cell maturation and may influence the outcome of acute infection.

## Introduction

Hepatitis C viral (HCV) infection is known for its high propensity to establish persistent infection [1]. Despite the advent of highly effective direct anti-viral treatment options, complications of cirrhosis related to chronic HCV will continue to increase [2]. The host immune response early in HCV infection is thought to determine subsequent outcome [3], suggesting an important role for innate immunity in viral elimination either directly, preventing establishment of infection, or indirectly, through priming of antigen-specific adaptive immune mechanisms [4].

Natural killer (NK) cells provide a major component of the innate antiviral immune response through recognition and killing of virally infected cells and induction of appropriate T cell responses [5]–[7] [8], [9]. Recent studies have highlighted important roles for NK cells in immunity against hepatotrophic viruses including HCV [10]. Several studies suggest that defective NK cell responses contribute to chronic HCV persistence [11]–[18]. It is not clear if HCV-related NK cell defects are simply a reflection of chronic inflammation in the setting of long-term antigen exposure as little is known of NKs in the acute setting and studies to date have yielded conflicting results [19]–[24].

In humans, relative expression of CD56 identifies functionally distinct immature/regulatory (CD56^bright^) and effector (CD56^dim^) NK cell subsets [8], [25]. An increased proportion of the CD56^bright^ NK subset has been reported in patients with chronic [15], [26], [27] and acute [23] HCV infection. NK cell activity is stringently controlled by inhibitory NK receptors (NKRs) which override signals provided by engagement of activating receptors [28]. NKRs include the predominantly inhibitory killer immunoglobulin-like receptors (KIR); C-type lectin-like receptors of the CD94/NKG2 family comprising inhibitory (NKG2A) and activatory (NKG2C/D) isoforms, as well as the natural cytotoxicity receptors (NCRs) such as NKp30, NKp44 and NKp46 receptors that deliver activatory signals [28], [29]. Dysregulation of NKR expression has been implicated in chronic viral persistence [15]–[17], [30], however, little is known of NKR expression on NK cells in acute HCV infection and these studies have produced conflicting results [22]–[24]. Studies to date suggest direct involvement of NKs in the acute phase of HCV infection; NK cell activation and phenotypic alterations have clearly been demonstrated [22]–[24]. Activation of NK cells early in HCV infection may favor induction and priming of downstream T cell responses and HCV clearance [24], [31].

Evidence for NK cell involvement in determining the outcome of HCV infection comes from studies that have attributed successful IFN-α therapy to rescue of NK cell function in patients chronically infected with HCV [11], [21], [32]–[34]. In addition, NK cell activity can be directly inhibited by HCV envelope [35], [36] and indirectly by core proteins [37], [38], suggesting that HCV immune evasion strategies may specifically target this cell population. The presence of circulating core in HCV-infected patients [39] makes it a good candidate for suppression of immune cells, which may not be directly infected, an interaction known to inhibit T cell responses [40]. Indeed, HCV-core protein has been shown to hamper dendritic cell (DC) maturation [39], directly suppress T cell activation [41] and to inhibit NK cell cytotoxicity indirectly through up-regulation and stabilization of MHC Class I molecules [37], [38].

Taken together, these studies strongly suggest that failure to eliminate HCV and development of a chronic course of infection may result from impaired NK cell level or function, furthermore, that HCV-core protein may be involved in NK cell inhibition. In the present study we explore the hypothesis that activation of peripheral NK cell populations is important for early control of virus and thus would predict outcome of acute HCV infection. In our cohort of patients acutely infected with HCV, we examine the levels, phenotype and function of NK cells, early and late in the course of acute infection, directly *ex vivo* and their relationship to subsequent outcome of viral infection and circulating levels of HCV-core. We also comprehensively explore the direct effect of extracellular core protein on NK cells.

## Results

### Levels of NK subsets in acute HCV infection

**Figure 1:**
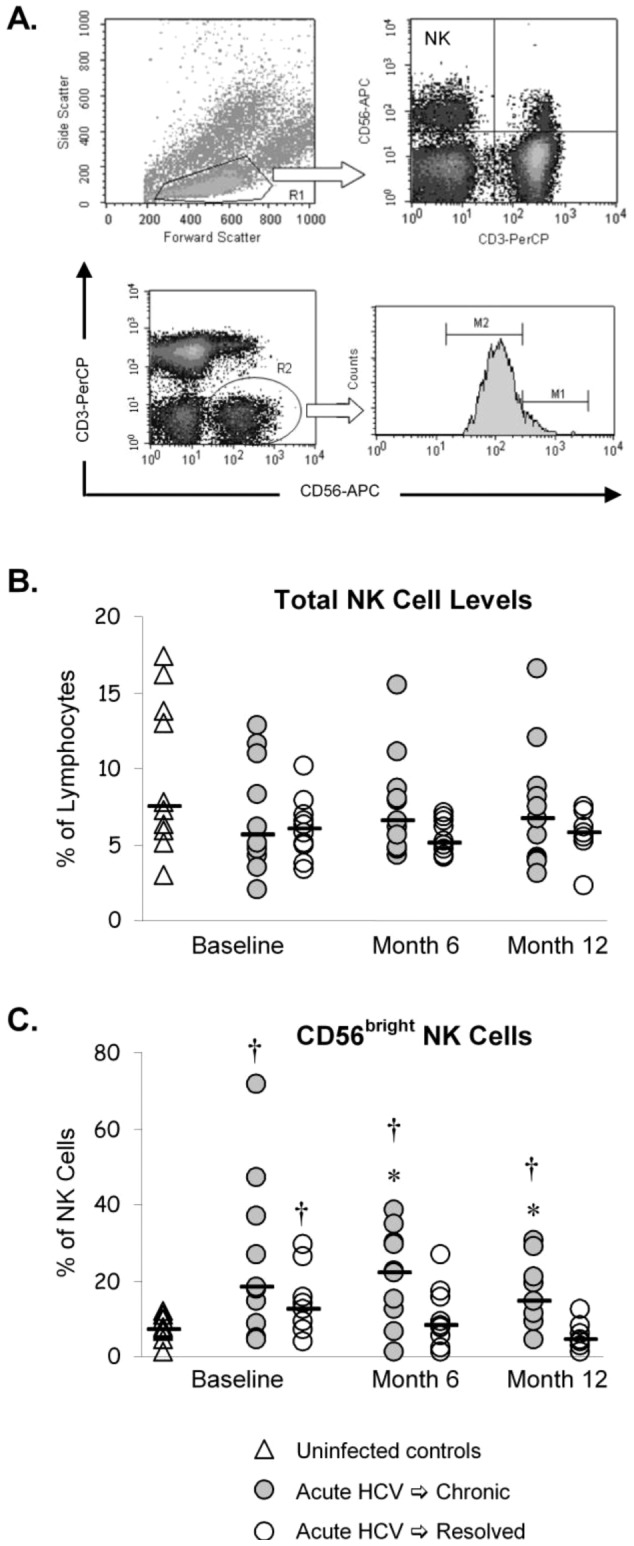
Frequency of NK cells and the immature/regulatory CD56^bright^ subset in acute HCV infection. **A)** Multi-parameter flow cytometric analysis was used to determine if the levels of peripheral NK cells (CD56^+^CD3^-^) were altered in acute HCV infection. **B)** Levels of total NK cells at time of enrollment and at six and twelve month follow up time points are similar to those detected in normal uninfected controls irrespective of subsequent clinical outcome. **C)** In contrast, an increase in the proportion of NK cells displaying a CD56^bright^ immature/regulatory phenotype was observed at baseline in acute HCV infection *per se* with a trend that was more pronounced in chronically evolving infection compared to patients who subsequently resolved infection. The relatively high proportion of NK cells with an immature/regulatory phenotype was maintained in the chronically evolving cohort and returned to normal levels in the spontaneously resolving patient group at six and twelve months post enrollment. * p<0.05 chronic versus resolved; † p<0.05 versus uninfected control (MWU).

Multi parameter flow cytometric analysis was used to determine if the levels of peripheral NK (CD56^pos^CD3^neg^) cells (**Figure 1A**) were altered in chronically evolving and/or spontaneously resolving acute HCV infection. As shown in **Figure 1B**, levels of NK cells at time of enrollment were similar to those detected in normal uninfected controls irrespective of subsequent outcome. In contrast, analysis of CD56^bright^ immature/regulatory and CD56^dim^ effector NK subsets revealed a significant increase in the immature/regulatory subset early in acute infection independent of subsequent outcome. Immature/regulatory NKs returned to normal levels in the spontaneously resolving but remained elevated in the chronically evolving patient group at six and twelve months post enrollment (**Figure 1C**). To assess if the increased proportion of CD56^bright^ NK cells was due to activation-induced expression of CD56 on the CD16^+^CD56^dim^ effector population, we analyzed the proportion of CD56^bright^ cells in the CD16^+^ NK cell subset. Patients and controls had similar levels of CD56^bright^ NK cells within the CD16^+^ NK cell population at all time-points (data not shown). These results suggest that the increase in CD56^bright^ NKs represents a true increase in the immature/regulatory subset and are consistent with the observation that the CD56^bright^/CD56^dim^ ratio is perturbed in chronic [15], [20], [26] and acute [23] HCV.

### Phenotype of NK cells in acutely infected patients

**Figure 2:**
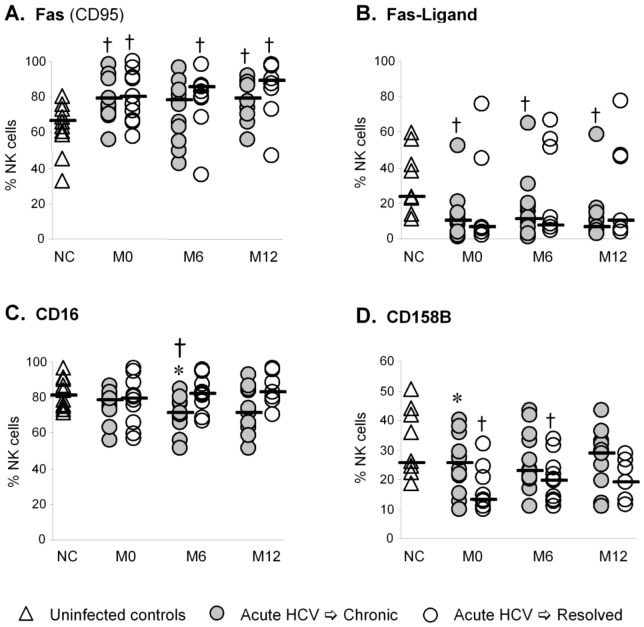
Phenotype of NK cells in acute HCV infection. Multi-parameter flow cytometric analysis was used to determine if the phenotype of NK cells was altered in acute HCV infection. Upregulation of Fas (**A**) and downregulation of Fas-Ligand (**B**) suggest activation of NK cells in acute HCV infection. Fas remains upregulated even after viral clearance. The decrease observed for NK CD16 expression in the chronically evolving patient group at month 6 (**C**) is consistent with altered NK subset distribution. Decreased expression of the inhibitory KIR CD158B (**D**) in the spontaneously resolving group at baseline suggests a genetic contribution to the outcome of acute HCV infection. * p<0.05 chronic versus resolved; † p<0.05 versus uninfected control (MWU) Abbreviations: NC = normal control; M = month.

Chronic viral infections including HCV can modulate expression of inhibitory and activating NKRs which control the activity of NK cells [16], [27], [30], [42]. However, little is known of the expression of these receptors in the acute setting; therefore, we examined the phenotype of NK cells in our patient cohort to determine whether altered NKR expression was associated with outcome of acute HCV infection. While the pattern of TRAIL, Fas (CD95) and Fas-Ligand (CD95-L) expression on NK cells (**Figure 2A and 2B**) suggest activation of NK cells in the acute phase of HCV infection *per se*, the phenotype of NK cells in the spontaneously resolving patient cohort was remarkably similar to the chronically evolving patient group (**Table 1**). Although our analysis did not reveal a distinctive pattern of NKR expression that predicted outcome, differential CD16 expression later (6 months post enrollment) in acute infection (**Figure 2C**) is consistent with sustained increases in the CD56^bright^ NK cell subset in chronically evolving HCV (**Figure 1C**). Lower levels of CD158B (KIR) in the spontaneously resolving cohort at baseline (**Figure 2D** **)** lends support to the hypothesis that genetically controlled inhibitory NK cell expression plays a role determining antiviral immunity and that diminished inhibitory responses may contribute to protection against HCV [43].

**Table 1:** Phenotype of natural killer (NK) cells in acute infection over time.

NKR	Normal (n = 10)	M0 Ch (n = 12)	M0 Re (n = 9)	M6 Ch (n = 12)	M6 Re (n = 10)	M12 Ch (n = 11)	M12 Re (n = 7)
CD158A	13.85 (5.55–31.53)	14.44 (1.65–41.81)	12.89 (0.10–26.21)	15.19 (1.53–25.02)	12.81 (0.18–23.37)	12.634 (0.96–27.3)	19.12 (0.02–24.08
CD158E	13.23 (0–22.76)	16.14 (0–38.92)	6.73 (0–19.86)	15.83 (0–37.62)	12.66 (0–22.41	17.26 (0–38.77)	7.89 (0–12.33)
NKG2A	50.02 (26.78–66.15)	52.31 (33.91–81.67)	56.31 (33.91–81.67)	50.58 (29.23–88.27)	45.72 (37.08–74.33)	50.06 (30.33–83.42)	47.87 (40.20–73.03)
NKG2C	11.22 (2.44–41.33)	7.14 (2.79–13.83)	7.7 (4.07–12.23)	8.61 (2.94–12.33)	8.46 (3.86–17.28)	6.42 (2.3–12.07)	6.21 (3.74–25.12)
NKG2D	97.96 (91.22–99.37)	94.27 (63.62–98.38)	93.03 (49.48–98.71)	93.38 (77.75–98.94)	95.29 (83.24–98.12)	93.91 (75.9–97.98)	95.4 (82.05–98.28)
NKp30	53.96 (42.03–84.72)	66.43 (31.27–83.59)	59.62 (38.33–74.89)	66.01 (39.74–89.84)*	55.45 (33.98–73.5)*	58.97 (35.55–83.21)*	46.78 (36.79–56.24)*
NKp44	6.07 (4.53–15.02)	5.81 (2.19–23.16)	4.92 (1.27–25.81)	5.29 (2.61–9.99)	5.64 (1.59–21.66)	4.44 (2.21–26.31)	3.72 (1.15–13.78)
NKp46	90.03 (73.21–96.71)	79.5 (56.6–96.48)	84.64 (75.62–94.32)	81.05 (31.63–97.34)	84.42 (65.19–94.04)	76.33 (56.66–94.06)	87.08 (61.45–92.99)
TRAIL	2.2 (0.6–7.07)	4.07 (2.11–12.56)†	3.17 (1.16–4.9)	5.41 (1.48–15.27)†	3.31 (1.5–8.46)†	3.36 (1.87–7.45)†	5.19 (3.78–10.45)†
CD94	73.28 (36.9–87.83)	75.47 (47.34–95.37)	72.9 (64.04–88.98)	74.32 (53.03–91.52)	70.77 (59.94–87.04)	71.84 (57.69–90.95)	74.07 (40.34–85.75)
CD161	45.10 (13.92–87.5)	45.76 (17.35–80.52)	48.02 (17.6–62.22)	42.3 (34.23–72.79)	47.07 (21.21–72.76)	46.41 (11.09–78.63)	51.62 (26.69–72.74)

Abbreviations: NKR = NK Receptor; M = Month; Ch = Chronic; Re = Resolved; † = p<0.05 versus normal control; * = p<0.05 Ch vs Re.

### Cytolytic activity of NK cells in acutely infected patients

**Figure 3:**
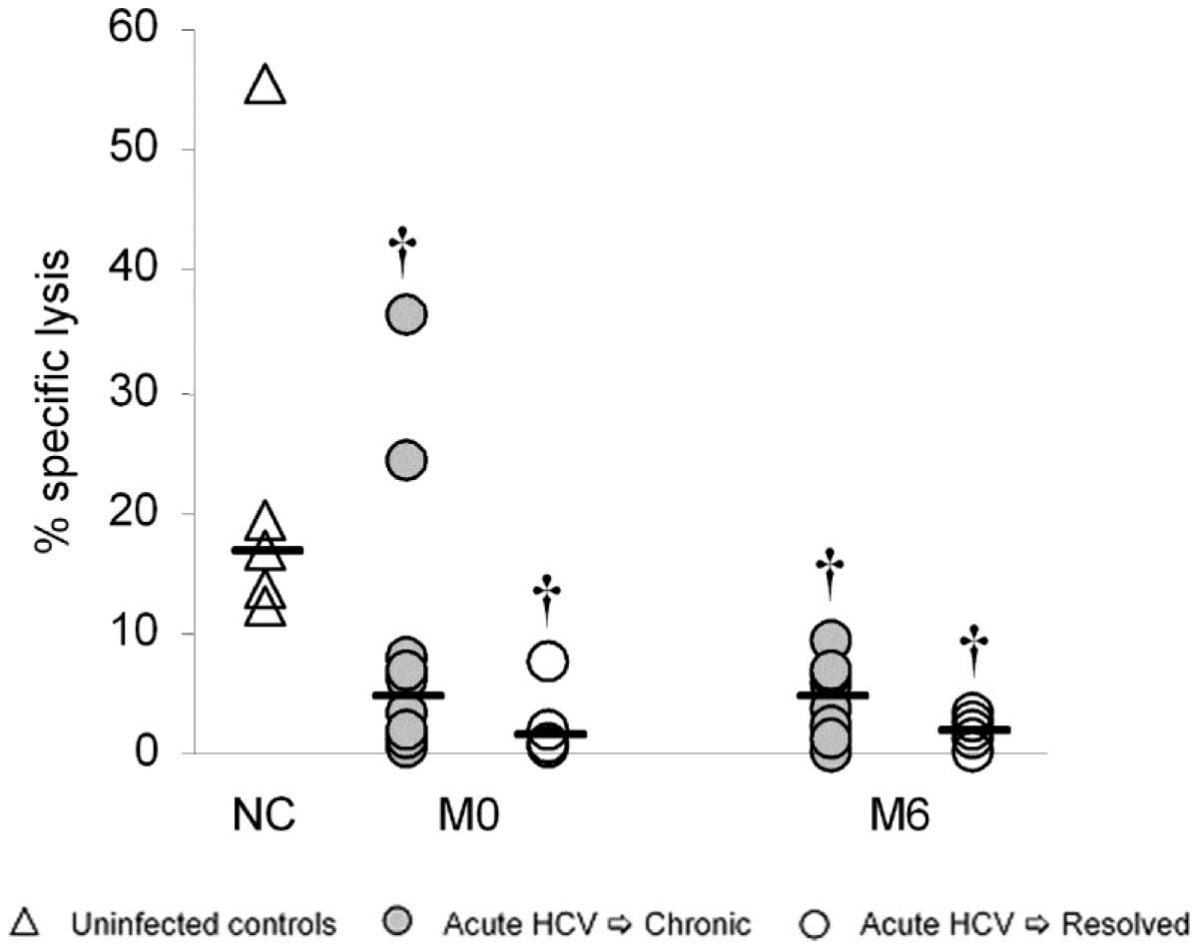
Functional competence of NK cells in acute HCV infection. Natural Cytotoxicity of bead-purified NK cells is compromised both early (M0) and late (M6) in acute HCV infection irrespective of outcome. * p<0.05 chronic versus resolved; † p<0.05 versus uninfected control (MWU) Abbreviations: NC = normal control; M = month.

We have previously reported that the IL-2 induced lymphokine activated killing (LAK) activity of isolated CD56^pos^ T cells is compromised in acute HCV patients who subsequently develop persistent infection but not in patients who spontaneously clear their infection [44]. In this study, we examine the capacity of NK cells to kill NK-sensitive target cells (K562s) in the absence of cytokine stimulation (natural cytotoxicity). Natural cytotoxicity is decreased in both the spontaneously resolving and chronically evolving HCV-infected patient groups compared to normal uninfected controls at baseline, and surprisingly, does not return to normal levels early (6 months post enrollment) after viral clearance (**Figure 3**).

### Effect of HCV-core protein on NK cells: phenotype and gene array

**Figure 4:**
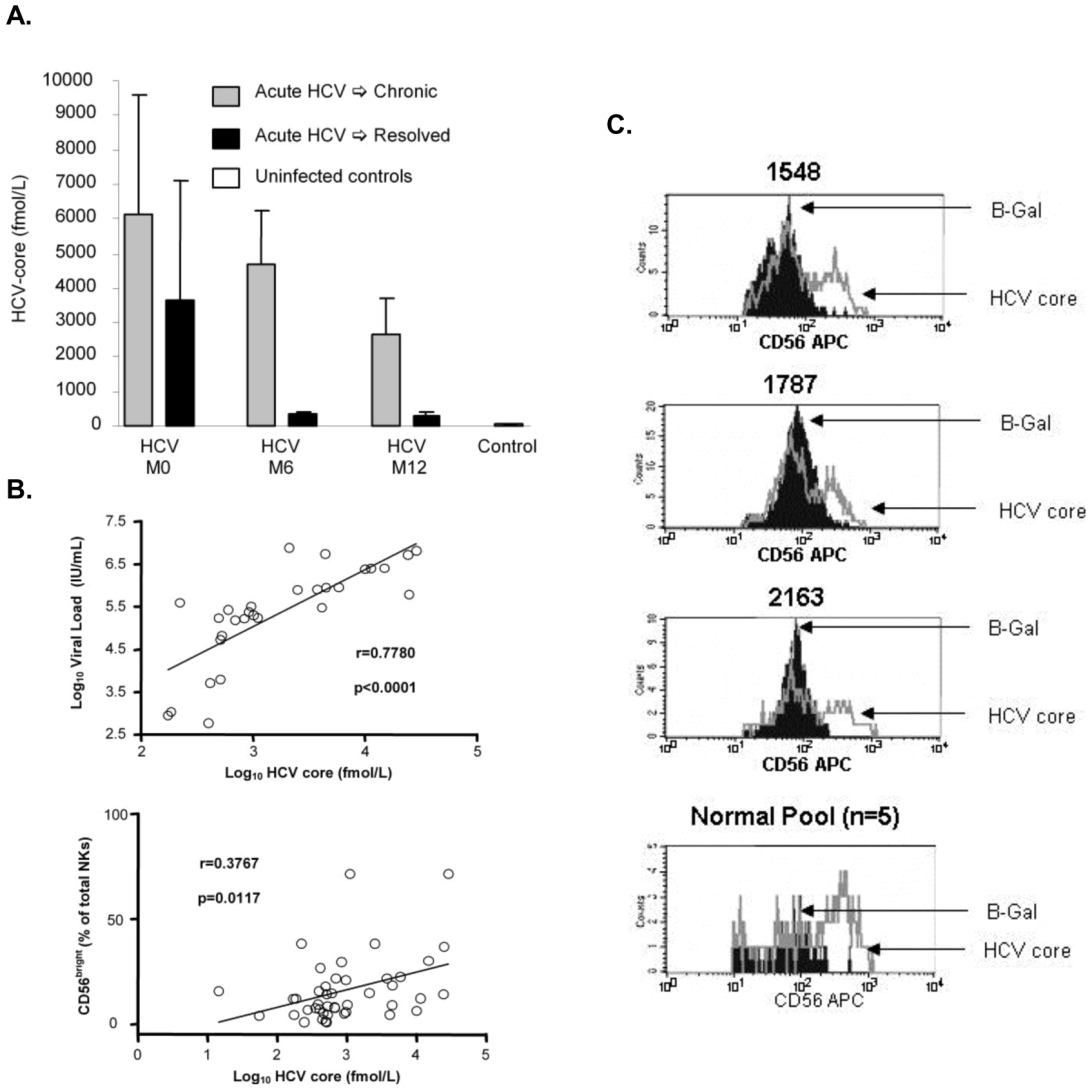
Circulating HCV-core levels in acute HCV infection and effect of extracellular core on NK cell CD56 expression. Circulating HCV-core protein levels as measured by ELISA **(A)**. As expected, core levels correlated directly with viral levels in HCV-infected patients. There was also a direct statistically significant correlation between core and the levels of CD56^bright^ NK cells in these patients. Multiple time points were used for chronic patients **(B)**. Incubation of highly purified FACS sorted CD56^bright^ NK cells, from normal uninfected control subjects, in the presence of HCV-core or beta-galactosidase (β-Gal) control protein demonstrated inhibition of CD56 down-regulation by exogenous HCV-core protein. Histograms of CD56 expression levels after incubation are shown for four individual experiments **(C)**. The first three histograms represent individual normal control subjects and the fourth histogram is derived from an experiment in which PBMCs from five normal uninfected control subjects were pooled prior to sorting on CD56^bright^ NK cells and incubation with HCV-core.

Having shown that acute infection was associated with an increased proportion of NK cells demonstrating an immature/regulatory phenotype (CD56^bright^) when analyzed directly *ex vivo*, we explored the possibility that specific HCV proteins might be involved. The direct effect of extracellular core on NK cells has not previously been investigated although HCV-core protein inhibition of NK cells through effects on MHC Class I have been reported [37], [38]. We therefore asked if circulating core contributed to the altered properties of NK cells that we had observed in our acutely infected patient cohort. We measured HCV-core protein levels in serum and assessed the effect of extracellular core on CD56 expression on the CD56^bright^ NK cell subset. As expected, core protein was detectable in serum from the acutely infected patient cohort at baseline (**Figure 4A**), and levels of circulating core protein correlated strongly with viral load. Remarkably, a correlation between circulating HCV-core levels and the proportion of peripheral NK cells with a CD56^bright^ immature/regulatory phenotype was also demonstrated (**Figure 4B**). We therefore FACS-sorted normal control peripheral NK cells into CD56^bright^ and CD56^dim^ sub-populations and assessed the effect of extracellular core on NKR and CD56 expression following stimulation with IL-15. Incubation with extracellular core protein maintained a significant proportion of the CD56^bright^ NK cell subset in an immature/regulatory phenotype compared to control protein (**Figure 4C**); however, it had no significant effect on NKR expression in the CD56^dim^ effector population (data not shown).

**Figure 5:**
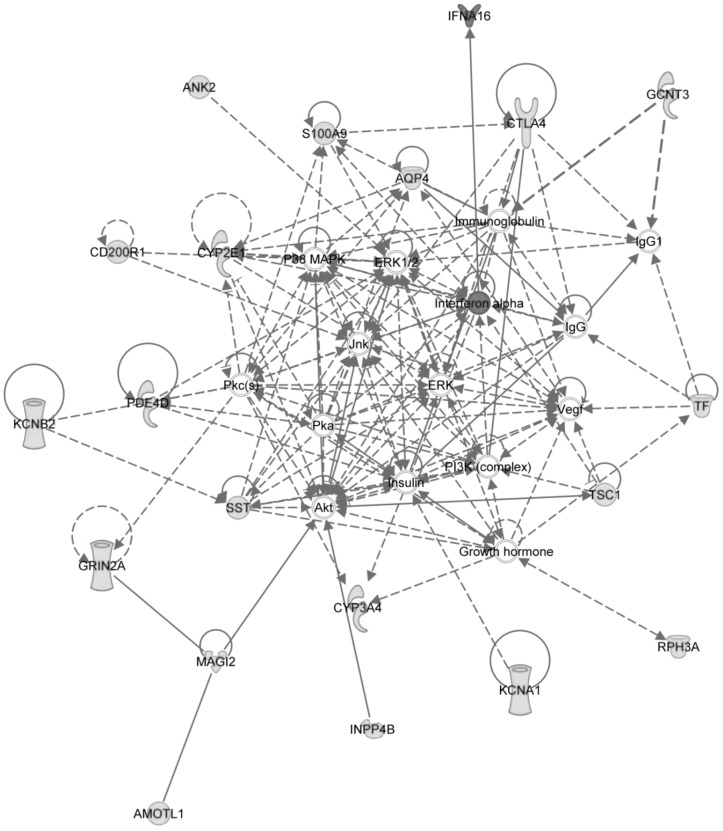
Network of differentially expressed genes. This network contains interconnections among 20 differentially expressed genes in response to HCV-core protein stimulation. Upregulated genes are indicated in green, and downregulated genes and gene classes in red. These differentially expressed genes include the cytotoxic T lymphocyte associated protein CTLA4, the cytochrome P450 proteins CYP2E1 and CYP3A4, the potassium voltage gated proteins KCNA1 and KCNB2, and the cell surface receptor CD200R1.

In order to comprehensively explore the effect of HCV-core protein on NK cells, we performed microarray on HCV-core-treated NK cells. Microarray analysis revealed differential expression among 121 genes, with a p-value less that 0.05 and greater than 2 fold change (**Table S1**). Remarkably, of these 121 differentially expressed genes, 115 genes were up-regulated and 6 were down-regulated in response to HCV-core stimulation. Among the most significantly up-regulated proteins, C8B, the complement component 8 protein, was up-regulated over 5 fold during HCV-core stimulation. In order to examine the differentially-expressed genes within the context of protein networks, we utilized Ingenuity Pathway Analysis (IPA). The highest scoring IPA network contained connections among 20 of the differentially expressed genes (**Figure 5**). Most of these genes are up-regulated in response to HCV-core protein, as indicated in green, aside from the Interferon alpha 16 (IFN-α16) cytokine which is down-regulated, as indicated in red. One of the central nodes, represented as IFN-α, represents a large class of IFN-α proteins, is indicated in red here due to the down-regulation of the IFN-α16 class member, the only member to exhibit differential expression. Connections between genes in this network are established by a variety of methods including co-citation in the literature, protein interactions, pathway members, activation interactions, and other biological relationships as assigned by IPA. Other members of the network include the cytotoxic T lymphocyte associated protein CTLA4, the cytochrome P450 proteins CYP2E1 and CYP3A4, the potassium voltage-gated proteins KCNA1 and KCNB2, and the cell surface receptor CD200R1. Down-regulation of the IFN-α16 gene in response to HCV-core was confirmed by PCR (n = 4, data not shown). These data suggest that HCV-core protein attenuates NK interferon type I responses.

## Discussion

Observations in chronic HCV infection for the most part suggest that long-term exposure to virus alters the phenotype and behavior of NK cells [12], [13] [15]–[18] [26] [27] [30] [43]. In the acute phase of infection, NK cells are activated although the precise role they play is unclear, as studies have provided conflicting data on the phenotype and function of NK cells in acute HCV infection [19]–[24]. In the present study, we have characterized NK cell level, subset distribution, phenotype and function both early (baseline) and late (6 and 12 months post enrollment) in acute HCV to evaluate their possible role in determining subsequent outcome of infection.

We demonstrate the overall levels of NK cells are not affected by acute HCV infection. A significant alteration in NK cell subset distribution has previously been reported for chronic [15], [26] [27], and acute [23] HCV infection. Our data show that expansion in immature/regulatory CD56^bright^ NK cells is an early event in acute HCV infection which normalizes upon viral clearance. We also show that NKR expression on NK cells was remarkably stable. Subtle changes in the pattern of NKR expression are suggestive of activation of this population upon encounter with the virus; however, distinctive patterns do not correlate with development of persistent infection or spontaneous viral clearance. Phenotypic alterations of NK cells in acute HCV infection have been reported but are difficult to interpret. Amadei *et al*., observed an increased expression of NKG2D on NK cells, irrespective of the outcome, as compared with healthy controls which is consistent with activation [23]. Alter *et al*., showed that NK cells from acute infected patients demonstrated lower frequencies of NKp46- and NKp30-expressing NK cells and these lower levels correlated with HCV clearance [22]. This finding is somewhat counterintuitive as high levels of NKp30 [45] and NKp46 [19] expression have recently been associated with protection against HCV infection in exposed uninfected individuals and NKp46 expression correlates with anti-HCV activity *in vitro* [42], [46], [47]. The authors suggest that activation-induced down-regulation of NCRs may account for the diminished percentage of NK cells expressing NKp46 and NKp30 in patients who resolve acute infection and may reflect that early NK cell activation results in the onset of an effective innate immune response that participates in viral clearance [22]. In our study, we observed a relative increase in the stimulatory NKp30 NCR in the later stages of acute infection (6 and 12 months after enrollment) in the chronically evolving relative to the spontaneously resolving patient cohort which is consistent with continued activation of NK cells in the presence of virus. We did not find these NCRs to be significantly differentially expressed on NK cells compared to normal controls either in the early or late stages of acute HCV infection. It is possible that the peripheral NK cell population is responding continually to chronic inflammation and constantly in flux. Once a KIR gene is expressed by a T cell clone or NK cell, it is stably maintained in the progeny of these cells [48]. At baseline, the acute patient cohort who subsequently resolve their infection have significantly lower levels of inhibitory CD158B expressing NK cells suggesting that diminished inhibitory responses upon initial encounter with HCV may contribute to protection against HCV and that these responses may in part be genetically controlled [43]. Further studies using well defined cohorts of patients with acute HCV infection are needed to define the contributions of individual NKRs to resolution.

While we observed altered NK cell subset distribution and subtle changes in NKR expression by NK cells in the acute phase of HCV infection that correlated with subsequent outcome of infection, these did not translate into dramatic functional consequences. Natural cytotoxicity (NC), cytolytic activity in the absence of cytokine activation, a property attributed to the CD56^dim^ mature NK cell subset [8], was compromised at baseline in keeping with the increased proportion of immature/regulatory CD56^bright^ NK cells at this time in the acutely infected patient populations. Other studies have reported increased or unchanged degranulation/cytotoxicity in acute infection [22]–[24]; these differences may be explained by the use of unfractionated peripheral blood mononuclear cells and different methods of activation. Of note, we did not see any differences in IL-15 stimulated killing activity in our patient cohort compared to controls (data not shown). Surprisingly, natural cytotoxicity did not recover upon viral clearance in the spontaneously resolving patient cohort although in this group NK cell subset distribution had returned to normal by that time. This suggests that altered subset distribution alone does not account for the reduction in NC and there may be sustained inhibition of the mature NK cell cytolytic activity. Taken together, these data suggest that acute HCV infection is associated with an accumulation of immature/regulatory NK cells and impaired natural cytotoxicity. Work is ongoing to define if these functions are differentially restored with direct-acting antiviral therapy in patients with chronic HCV infection.

The immunomodulatory properties of core [41] and its presence in the circulation of HCV-infected patients [39] makes it a good candidate for suppression of NK cells, which are likely not directly infected**.** We observed a direct correlation between the proportion of peripheral NK cells with a CD56^bright^ immature/regulatory phenotype in our patient cohort and quantities of circulating HCV-core. Morevoer, we demonstrate that *in vitro* incubation of CD56^bright^ NK cells with exogenous HCV-core protein maintains the immature/regulatory CD56^bright^ phenotype in a significant proportion of NK cells. HCV-core likely binds gC1qR expressed on NK cells as has been demonstrated for various innate and adaptive immune cell types [40], [41], [49]. We show for the first time a role for exogenous HCV-core in NK cell maturation. It is likely that exogenous HCV-core protein contributes to HCV immune evasion in a distinct manner from endogenously expressed or presented HCV-core protein [37], [38], [49]. In order to comprehensively explore the effect of HCV-core protein on NK cells, we performed microarray on HCV-core treated NK cells. The effects of extracellular core on isolated NK cells is subtle as evidenced by the relatively small number of genes that demonstrated a significant change compared to control treated cells. Remarkably, 115 of the 121 genes differentially expressed by HCV-core were upregulated and only 6 (<5%) were downregulated. Within the context of protein networks, we identified a network that contained 20 of the differentially expressed genes, all of which were upregulated with the exception of IFN-α16 suggesting that NK interferon type I responses may be negatively regulated by HCV-core protein.

In summary, NK cell subset distribution is perturbed early in acute HCV infection and returns to normal on viral clearance without normalization of natural cytotoxicity. Exogenous HCV-core protein may contribute to the maintenance of NK cells in the CD56^bright^ immature/regulatory state, likely through interaction with cell surface receptors including gC1qR, although the mechanism of HCV-core modulation of NK cells remains to be elucidated.

## Materials and Methods

### Ethics statement

The study protocol was approved by the Institutional Review Board at the University of Colorado Denver. Both written and oral consent was obtained before samples were collected.

### Study population

The study group comprised acutely HCV-infected patients recruited from multiple sites. Acute HCV was diagnosed based on: HCV Ab seroconversion in a subject with previously negative HCV testing; seroconversion in a subject with new-onset risk factors and ALT greater than 10-fold normal; or HCV RNA positivity with HCV Ab negativity. Twenty two treatment naïve patients (12 male and 10 female; mean age 37 years) were recruited for the present study. The majority of patients were Caucasian (90.9%). Spontaneous viral resolution (n = 10) and chronicity (n = 12) were defined as the absence or presence of HCV RNA at 6 months post enrollment with at least two viral determinations. Seventy seven percent of patients had known genotype-1 infection. The predominant risk factor was intravenous drug usage (50%) followed by sexual transmission (18.17%), surgery (13.63%) and needle stick injuries (9.1%). For two of the patients (both of which developed chronic infection) the risk factor for HCV acquisition was unknown.

### Sample preparation and storage

Peripheral blood and serum were drawn from the acutely infected cohort at baseline and 6 and 12 months later. Peripheral blood was drawn at a single time point from normal controls. Peripheral blood mononuclear cells (PBMCs) were isolated by Ficoll (Amersham Biosciences, Piscataway, NJ) density gradient centrifugation and cryopreserved for subsequent analyses. Plasma preparation tubes (PPT tubes, BD Biosciences, San Jose, CA) were used to isolate plasma from whole blood, which was frozen and later thawed for viral load and genotype testing. HCV genotyping (LiPA) and viral level determination (HCV RNA 3.0 bDNA, lower limit 615 copies/ml) were performed by Bayer Reference Testing Laboratory (Berkeley, CA).

### Flow cytometric analysis of cell surface antigens

Four-color multiparameter flow cytometry was performed using a BD FACSCalibur instrument (BD Biosciences, San Jose, CA) compensated with single fluorochromes and analysed using CellQuest software (BD Biosciences). Flurochrome-labeled (FITC/PE/PerCP/APC) monoclonal antibodies (MAbs) specific for CD3, CD4, CD8, CD56, CD161, CD94, CD16, CD158A, CD158B, CD158E (NKB1), and NKG2D were purchased from BD Biosciences. Anti-NKG2C-PE and TRAIL-PE MAbs were supplied by R&D systems (Minneapolis, MN). Anti-NKG2A-PE, NKp30-PE, NKp44-PE, and NKp46-PE were obtained from Immunotech (Beckman Coulter, Fullerton, CA). PBMCs (2.5×10^5^), were stained for cell surface antigen expression at 4°C in the dark for 30 minutes. Then, washed twice in 2 ml phosphate-buffered saline (PBS) containing 1% bovine serum albumin and 0.01% sodium azide (Facs Wash) and subsequently fixed in 200 µl of 1% paraformaldehyde (Sigma-Aldrich, St. Louis, MO). Isotype-matched fluorescently-labeled control antibodies were used to determine background levels of staining. Lymphocytes were identified by characteristic forward scatter (fsc) and side scatter (ssc) parameters and populations of interest were gated on positive staining for CD56 and negative staining for CD3 within the lymphocyte population (**Figure 1A**). Results are expressed as % positive of gated populations or as median fluorescent intensity (MFI) where appropriate.

### Cytotoxicity assays

Thawed mononuclear cell suspensions were enriched for NK cells using the NK Isolation Kit II from Miltenyi Biotec (Gladbach, Germany) according to the manufacturer's instructions. Median purity of NK cells was >90% in all cases. Following isolation, NK cells were cultured with or without IL-15 (25ng/ml, R&D systems, Minneapolis, MN) for 48 hours at 37°C and 5% CO_2_. Following culture carboxy fluorescein succinimidyl ester (CFSE) labeled target cells (K562s) were added to the NK populations at effector:target concentrations of 0∶1 (negative control) and 10∶1 and incubated at 37°C for 4 hours. Cytotoxicity was measured using the flow-cytometry based Total Cytotoxicity & Apoptosis Detection Kit from Immunochemistry (Bloomington, MN). Immediately before acquisition, 7-aminoactinomycin D (7-AAD) was added to effector:target populations and incubated for 15 minutes on ice. Cells treated with 0.1% Triton-X served as positive controls.

### HCV-core ELISA

Circulating HCV-core levels were quantified in serum collected at baseline (M0), and 6 (M6) and 12 months (M12) later from chronically evolving (n = 10) and spontaneously resolving (n = 8) acute HCV patients using the Ortho HCV-core Ag ELISA test kit (Wako Chemicals, Richmond, VA) following the manufacturer's protocol. Serum from five uninfected control subjects served as negative controls.

### NK subset isolation and culture with core protein

PBMCs were sterile sorted into immature/regulatory CD56^bright^ and effector CD56^dim^ subsets using a FACSAria cell sorter (BD Biosciences). Isolated NK cell subsets (CD56^bright/dim^) were diluted to 1×10^6^/ml in RPMI +10% human serum and incubated with 5 µg/ml HCV-core protein (β-gal-core) or control protein (β-gal, ViroGen, Watertown, MA) for 8 hours. Cells were then washed and incubated with IL-15 (25 ng/ml, R&D Systems) for 48 hours. Cells were harvested and analyzed by flow cytometry for CD56 expression level. The CD56^dim^ subsets were also phenotypically characterized as described above.

### Expression profiling of hepatitis C viral core protein stimulation

Isolated NK cells from three normal control subjects (1×10^6^/ml) were incubated for 1 hour with HCV-core or control (β-Gal) protein (5 ug/ml). Then, IL-15 (5 ng/ml) was added to all samples and NK cells were incubated for an additional 47 hours. Cells were harvested in TRIzol Reagent (Life Technologies, Grand Island, NY) and RNA extracted (following the manufacturer's procedure) for microarray analysis. Template RNA quality was assessed with the Agilent Bioanalyzer 2100 and an Agilent Nano RNA 6000 kit per the Agilent protocol. Processing and analyses were performed in Genespring GX 10 (Agilent Technologies, Santa Clara, CA). Differential expression was assayed (in triplicate for each individual subject) using Agilent 4x44K human microarrays according to the manufacturer's instructions (Agilent Technologies). The NK response to the HCV-core protein was determined by comparing the gene expression of cells stimulated with HCV-core versus the β-gal control protein. Genes that exhibited a statistically significant difference in expression between the two groups, as evaluated by a 0.05 p-value threshold and a 2 fold difference in gene expression were determined. Genes that were differentially expressed were then examined using the Ingenuity Pathway Analysis (IPA) (http://www.ingenuity.com/products/ipa) software in order to examine the differentially expressed genes within the context of cellular networks and to identify enriched functional classes among the differentially expressed genes.

### Statistical analyses

Results are expressed as median (range). Non-parametric Mann Whitney U was used to compare differences between patient groups and over time. Spearman was used for correlation analyses. Significance was defined as a p value of less than 0.05. The JMP 6.0 (SAS Institute, Inc, Cary NC) statistical package was used.

## Supporting Information

Table S1 **Provides a complete list of the 121 genes differentially expressed (with a p-value less that 0.05 and greater than 2 fold change) in response to HCV-core stimulation.**
(DOC)
