# Supplementary material for: Extracellular HCV-Core Protein Induces an Immature Regulatory Phenotype in NK Cells: Implications for Outcome of Acute Infection
Source: PLoS One. 2014 Jul 30;9(7):e103219. doi: 10.1371/journal.pone.0103219 (PMC4116173; doi:10.1371/journal.pone.0103219)
Supplement: Table S1 — Provides a complete list of the 121 genes differentially expressed (with a p-value less that 0.05 and greater than 2 fold change) in response to HCV-core stimulation. (DOC) [file pone.0103219.s001.doc]

| **Supplemental Table 1: Genes differentially regulated greater than 2 fold in natural killer (NK) cells**  **by HCV-core protein.** | | | | | | | | | | | | | | | | | | | |
| --- | --- | --- | --- | --- | --- | --- | --- | --- | --- | --- | --- | --- | --- | --- | --- | --- | --- | --- | --- |
|  | | | |  | | |  | | |  | | |  | |  | | | | |
| **ProbeName** | **p-value*** | | **Fold change†** | | | **Regulation** | | | **GeneSymbol** | | | **Description** | | | | | |  | |
| A_33_P3212052 | 0.0409 | | 6.57 | | | up | | |  | | | Putative uncharacterized protein ENSP00000339355 Fragment | | | | | | |  |
| A_33_P3423854 | 0.0464 | | 5.01 | | | up | | | C8B | | | complement component 8, beta polypeptide (C8B) | | | | | | |  |
| A_23_P212508 | 0.0306 | | 4.70 | | | up | | | TF | | | transferrin (TF) | | | | | | |  |
| A_33_P3242064 | 0.0333 | | 4.63 | | | up | | | OR5J2 | | | olfactory receptor, family 5, subfamily J, member 2 (OR5J2) | | | | | | |  |
| A_24_P58673 | 0.0137 | | 4.58 | | | up | | | REG4 | | | regenerating islet-derived family, member 4 (REG4), variant 2 | | | | | | |  |
| A_33_P3323599 | 0.0025 | | 4.50 | | | up | | | CCDC129 | | | cDNA FLJ38344 fis, highly similar to sperm specific antigen 2 | | | | | | |  |
| A_32_P70818 | 0.0158 | | 4.17 | | | up | | | PAX9 | | | paired box 9 (PAX9) | | | | | | |  |
| A_33_P3281745 | 0.0450 | | 4.10 | | | up | | |  | | | 17000600021474 GRN_PRENEU cDNA 5' | | | | | | |  |
| A_24_P456490 | 0.0133 | | 3.94 | | | up | | | C1orf204 | | | chromosome 1 open reading frame 204 (C1orf204) | | | | | | |  |
| A_32_P169114 | 0.0028 | | 3.58 | | | up | | | GRIN2A | | | glutamate receptor, ionotropic, N-methyl D-aspartate 2A (GRIN2A) | | | | | | |  |
| A_33_P3333238 | 0.0188 | | 3.43 | | | up | | | HOXD9 | | | homeobox D9 (HOXD9) | | | | | | |  |
| A_33_P3246997 | 0.0123 | | 3.37 | | | up | | | LUC7L3 | | | cisplatin resistance-associated overexpressed protein | | | | | | |  |
| A_23_P420209 | 0.0400 | | 3.35 | | | up | | | GCNT3 | | | glucosaminyl (N-acetyl) transferase 3, mucin type (GCNT3) | | | | | | |  |
| A_23_P23048 | 0.0309 | | 3.34 | | | up | | | S100A9 | | | S100 calcium binding protein A9 (S100A9) | | | | | | |  |
| A_24_P401768 | 0.0323 | | 3.32 | | | up | | | AMOTL1 | | | angiomotin like 1 (AMOTL1) | | | | | | |  |
| A_33_P3329023 | 0.0386 | | 3.29 | | | up | | | FAM69A | | | family with sequence similarity 69, member A (FAM69A) | | | | | | |  |
| A_23_P47885 | 0.0136 | | 3.25 | | | up | | | LRIG3 | | | leucine-rich repeats and immunoglobulin-like domains 3 (LRIG3) | | | | | | |  |
| A_33_P3866042 | 0.0499 | | 3.25 | | | up | | | D21S2090E | | | D21S2090E mRNA sequence | | | | | | |  |
| A_24_P388662 | 0.0381 | | 3.11 | | | up | | | C4orf35 | | | chromosome 4 open reading frame 35 (C4orf35) | | | | | | |  |
| A_33_P3364826 | 0.0415 | | 3.08 | | | up | | |  | | | armadillo repeat containing 4 | | | | | | |  |
| A_33_P3386746 | 0.0325 | | 3.01 | | | up | | | C10orf112 | | | MAM and LDL-receptor class A domain-containing protein C10orf112 | | | | | | |  |
| A_23_P12643 | 0.0153 | | 2.97 | | | up | | | AS3MT | | | arsenic (+3 oxidation state) methyltransferase (AS3MT) | | | | | | |  |
| A_23_P24543 | 0.0251 | | 2.97 | | | up | | | FAM55A | | | family with sequence similarity 55, member A (FAM55A) | | | | | | |  |
| A_33_P3227676 | 0.0480 | | 2.95 | | | up | | | C2orf72 | | | chromosome 2 open reading frame 72 (C2orf72) | | | | | | |  |
| A_33_P3391370 | 0.0025 | | 2.92 | | | up | | | OR10AG1 | | | olfactory receptor, family 10, subfamily AG, member 1 (OR10AG1) | | | | | | |  |
| A_33_P3637366 | 0.0306 | | 2.91 | | | up | | | LOC285547 | | | cDNA FLJ39049 fis, clone NT2RP7011132 | | | | | | |  |
| A_24_P273756 | 0.0231 | | 2.90 | | | up | | | TP63 | | | tumor protein p63 (TP63), transcript variant 1 | | | | | | |  |
| A_33_P3380529 | 0.0152 | | 2.86 | | | up | | | PRTFDC1 | | | phosphoribosyl transferase domain containing 1 (PRTFDC1) | | | | | | |  |
| A_24_P520767 | 0.0098 | | 2.85 | | | up | | | LOC149351 | | | hypothetical protein LOC149351 | | | | | | |  |
| A_33_P3224290 | 0.0116 | | 2.83 | | | up | | | OR4S2 | | | olfactory receptor, family 4, subfamily S, member 2 (OR4S2) | | | | | | |  |
| A_23_P18152 | 0.0167 | | 2.83 | | | up | | | ATP2B2 | | | ATPase, Ca++ transporting, plasma membrane 2 (ATP2B2), variant 1 | | | | | | |  |
| A_33_P3336282 | 0.0419 | | 2.81 | | | up | | | TAF4B | | | TAF4b RNA polymerase II, (TBP)-associated factor | | | | | | |  |
| A_23_P370054 | 0.0005 | | 2.76 | | | up | | | MAGEB18 | | | melanoma antigen family B, 18 (MAGEB18), mRNA [NM_173699] | | | | | | |  |
| A_33_P3314794 | 0.0279 | | 2.76 | | | up | | | IQCA1 | | | IQ motif containing with AAA domain 1 (IQCA1) | | | | | | |  |
| A_33_P3219865 | 0.0202 | | 2.75 | | | up | | | LOC400620 | | | clone IMAGE:3342755 | | | | | | |  |
| A_33_P3290011 | 0.0018 | | 2.75 | | | up | | | LHFPL3 | | | lipoma HMGIC fusion partner-like 3 (LHFPL3) | | | | | | |  |
| A_32_P526255 | 0.0479 | | 2.74 | | | up | | | PNPLA1 | | | patatin-like phospholipase domain containing 1 (PNPLA1) | | | | | | |  |
| A_24_P95723 | 0.0482 | | 2.73 | | | up | | | KIAA0125 | | | KIAA0125 (KIAA0125) | | | | | | |  |
| A_33_P3230995 | 0.0139 | | 2.67 | | | up | | |  | | | 1Z93_A Chain A, Human Carbonic Anhydrase | | | | | | |  |
| A_33_P3402188 | 0.0043 | | 2.67 | | | up | | | TAS2R19 | | | MSTP058 mRNA | | | | | | |  |
| A_33_P3287967 | 0.0302 | | 2.63 | | | up | | | ANK2 | | | ankyrin 2, neuronal (ANK2), transcript variant 1 | | | | | | |  |
| A_33_P3420757 | 0.0246 | | 2.63 | | | up | | | AQP4 | | | aquaporin 4 (AQP4), transcript variant a | | | | | | |  |
| A_32_P70056 | 0.0451 | | 2.57 | | | up | | | LOC642864 | | | spermatogenesis-related protein 7 mRNA | | | | | | |  |
| A_32_P420563 | 0.0408 | | 2.57 | | | up | | | RNF215 | | | ring finger protein 215 (RNF215) | | | | | | |  |
| A_23_P155463 | 0.0307 | | 2.56 | | | up | | | LRRC2 | | | leucine rich repeat containing 2 (LRRC2), transcript variant 1 | | | | | | |  |
| A_32_P89899 | 0.0271 | | 2.56 | | | up | | | GABRG1 | | | gamma-aminobutyric acid (GABA) A receptor, gamma 1 (GABRG1) | | | | | | |  |
| A_24_P83922 | 0.0175 | | 2.56 | | | up | | | SNRPC | | | small nuclear ribonucleoprotein polypeptide C (SNRPC), variant 1 | | | | | | |  |
| A_33_P3283083 | 0.0184 | | 2.55 | | | up | | | INPP4B | | | inositol polyphosphate-4-phosphatase, type II, 105kDa (INPP4B) | | | | | | |  |
| A_33_P3525067 | 0.0186 | | 2.52 | | | up | | | LOC285627 | | | hypothetical LOC285627 (LOC285627) | | | | | | |  |
| A_33_P3250740 | 0.0014 | | 2.50 | | | up | | | DCAF8L1 | | | DDB1 and CUL4 associated factor 8-like 1 (DCAF8L1) | | | | | | |  |
| A_23_P355405 | 0.0475 | | 2.50 | | | up | | | CNTNAP4 | | | contactin associated protein-like 4 (CNTNAP4), transcript variant 2 | | | | | | |  |
| A_23_P72668 | 0.0295 | | 2.49 | | | up | | | SDPR | | | serum deprivation response (SDPR) | | | | | | |  |
| A_33_P3248052 | 0.0371 | | 2.48 | | | up | | | NLGN4Y | | | neuroligin 4, Y-linked (NLGN4Y), transcript variant 2 | | | | | | |  |
| A_23_P82503 | 0.0149 | | 2.47 | | | up | | | PEG10 | | | paternally expressed 10 (PEG10), transcript variant 1 | | | | | | |  |
| A_33_P3373388 | 0.0439 | | 2.47 | | | up | | | GABRA1 | | | gamma-aminobutyric acid (GABA) A receptor, alpha 1 (GABRA1) | | | | | | |  |
| A_33_P3395562 | 0.0475 | | 2.46 | | | up | | | C1orf141 | | | chromosome 1 open reading frame 141 (C1orf141) | | | | | | |  |
| A_23_P361744 | 0.0324 | | 2.45 | | | up | | | ATXN3L | | | ataxin 3-like (ATXN3L) | | | | | | |  |
| A_33_P3383276 | 0.0436 | | 2.45 | | | up | | | OR6C2 | | | olfactory receptor, family 6, subfamily C, member 2 (OR6C2) | | | | | | |  |
| A_23_P57020 | 0.0106 | | 2.44 | | | up | | | GTSF1L | | | gametocyte specific factor 1-like (GTSF1L), transcript variant 2 | | | | | | |  |
| A_33_P3239644 | 0.0157 | | 2.44 | | | up | | | LOC645188 | | | cDNA clone IMAGE:4838974 | | | | | | |  |
| A_23_P48229 | 0.0328 | | 2.43 | | | up | | | KCNA1 | | | potassium voltage-gated channel, shaker-related subfamily (KCNA1) | | | | | | |  |
| A_23_P356667 | 0.0194 | | 2.43 | | | up | | | CCDC83 | | | coiled-coil domain containing 83 (CCDC83) | | | | | | |  |
| A_33_P3308686 | 0.0239 | | 2.42 | | | up | | | CC2D2A | | | coiled-coil and C2 domain containing 2A (CC2D2A) | | | | | | |  |
| A_33_P3208951 | 0.0049 | | 2.39 | | | up | | | OR51L1 | | | olfactory receptor, family 51, subfamily L, member 1 (OR51L1) | | | | | | |  |
| A_32_P470868 | 0.0407 | | 2.37 | | | up | | | FAM194B | | | family with sequence similarity 194, member B (FAM194B) | | | | | | |  |
| A_24_P841677 | 0.0263 | | 2.36 | | | up | | | CXorf23 | | | chromosome X open reading frame 23 (CXorf23) | | | | | | |  |
| A_33_P3338484 | 0.0278 | | 2.36 | | | up | | |  | | | DKFZp686M06185_r1 686 (synonym: hlcc3) | | | | | | |  |
| A_33_P3247027 | 0.0266 | | 2.35 | | | up | | | LOC199897 | | | PREDICTED: hypothetical LOC199897 (LOC199897) | | | | | | |  |
| A_24_P211797 | 0.0230 | | 2.34 | | | up | | | ICA1L | | | islet cell autoantigen 1,69kDa-like (ICA1L), transcript variant 2 | | | | | | |  |
| A_33_P3418165 | 0.0335 | | 2.34 | | | up | | | LOC729289 | | | PREDICTED: hypothetical LOC729289 (LOC729289) | | | | | | |  |
| A_33_P3358397 | 0.0195 | | 2.34 | | | up | | | LGI2 | | | leucine-rich repeat LGI family, member 2 (LGI2) | | | | | | |  |
| A_24_P558750 | 0.0161 | | 2.33 | | | up | | | hCG_1808463 | | | hypothetical protein LOC100132963 (LOC100132963) | | | | | | |  |
| A_23_P138168 | 0.0284 | | 2.33 | | | up | | | CNN3 | | | calponin 3, acidic (CNN3) | | | | | | |  |
| A_33_P3368201 | 0.0251 | | 2.30 | | | up | | | LOC732096 | | | cDNA clone IMAGE:4822577 | | | | | | |  |
| A_33_P3310293 | 0.0353 | | 2.28 | | | up | | | PKIG | | | protein kinase (cAMP-dependent, catalytic) inhibitor gamma (PKIG) | | | | | | |  |
| A_24_P109071 | 0.0396 | | 2.28 | | | up | | | KIF27 | | | kinesin family member 27 (KIF27) | | | | | | |  |
| A_33_P3228499 | 0.0351 | | 2.27 | | | up | | |  | | |  | | | | | | |  |
| A_33_P3213822 | 0.0489 | | 2.25 | | | up | | | KCNK2 | | | potassium channel, subfamily K, member 2 (KCNK2) | | | | | | |  |
| A_33_P3401556 | 0.0022 | | 2.24 | | | up | | | CTLA4 | | | cytotoxic T-lymphocyte-associated protein 4 (CTLA4) | | | | | | |  |
| A_23_P215214 | 0.0228 | | 2.24 | | | up | | | LMBR1 | | | limb region 1 homolog (mouse) (LMBR1) | | | | | | |  |
| A_24_P247849 | 0.0410 | | 2.24 | | | up | | |  | | | Sequence 1700 from Patent EP1308459 | | | | | | |  |
| A_33_P3745164 | 0.0118 | | 2.22 | | | up | | | CADM2 | | | cell adhesion molecule 2 (CADM2), transcript variant 1 | | | | | | |  |
| A_33_P3410123 | 0.0445 | | 2.21 | | | up | | | KIAA1751 | | | KIAA1751 (KIAA1751) | | | | | | |  |
| A_33_P3587611 | 0.0046 | | 2.20 | | | up | | | RPL36AP33 | | | cDNA clone MGC:23914 IMAGE:4769647 | | | | | | |  |
| A_33_P3401452 | 0.0035 | | 2.19 | | | up | | | CHD6 | | | chromodomain helicase DNA binding protein 6 (CHD6) | | | | | | |  |
| A_23_P41217 | 0.0357 | | 2.19 | | | up | | | CD200R1 | | | CD200 receptor 1 (CD200R1), transcript variant 1 | | | | | | |  |
| A_32_P85676 | 0.0364 | | 2.18 | | | up | | | STK32B | | | serine/threonine kinase 32B (STK32B) | | | | | | |  |
| A_23_P253542 | 0.0015 | | 2.17 | | | up | | | SMPX | | | small muscle protein, X-linked (SMPX) | | | | | | |  |
| A_33_P3359373 | 0.0004 | | 2.16 | | | up | | | TSC1 | | | cDNA clone CS0DD001YO24 of Neuroblastoma Cot 50-normalized | | | | | | |  |
| A_33_P3216207 | 0.0128 | | 2.16 | | | up | | |  | | |  | | | | | | |  |
| A_24_P12690 | 0.0122 | | 2.15 | | | up | | | IDO2 | | | indoleamine 2,3-dioxygenase 2 (IDO2) | | | | | | |  |
| A_23_P252817 | 0.0216 | | 2.14 | | | up | | | SST | | | somatostatin (SST) | | | | | | |  |
| A_23_P76234 | 0.0426 | | 2.13 | | | up | | | RPH3A | | | rabphilin 3A homolog (mouse) (RPH3A), transcript variant 1 | | | | | | |  |
| A_24_P344516 | 0.0466 | | 2.12 | | | up | | | ZNF702P | | | zinc finger protein 702 (pseudogene) (ZNF702P) | | | | | | |  |
| A_33_P3233666 | 0.0118 | | 2.12 | | | up | | | RPP30 | | | ribonuclease P/MRP 30kDa subunit (RPP30), transcript variant 1 | | | | | | |  |
| A_24_P170403 | 0.0343 | | 2.11 | | | up | | | C2orf78 | | | chromosome 2 open reading frame 78 (C2orf78) | | | | | | |  |
| A_24_P394940 | 0.0449 | | 2.11 | | | up | | | CYP2E1 | | | cytochrome P450, family 2, subfamily E, polypeptide 1 (CYP2E1) | | | | | | |  |
| A_33_P3244793 | 0.0231 | | 2.10 | | | up | | | GADL1 | | | glutamate decarboxylase-like 1 (GADL1) | | | | | | |  |
| A_33_P3329522 | 0.0437 | | 2.10 | | | up | | | LRRC17 | | | leucine rich repeat containing 17 (LRRC17), transcript variant 1 | | | | | | |  |
| A_33_P3240552 | 0.0414 | | 2.09 | | | up | | | PDE4D | | | cDNA, FLJ97311 | | | | | | |  |
| A_33_P3285799 | 0.0093 | | 2.08 | | | up | | | AKD1 | | | cDNA FLJ16163 fis, clone BRCAN2014229 | | | | | | |  |
| A_32_P197621 | 0.0361 | | 2.07 | | | up | | | GEMIN8P4 | | | gem associated protein 8 pseudogene 4 (GEMIN8P4) | | | | | | |  |
| A_33_P3410259 | 0.0500 | | 2.07 | | | up | | | hCG_2045710 | | | hypothetical LOC100133545 (LOC100133545) | | | | | | |  |
| A_33_P3317109 | 0.0087 | | 2.07 | | | up | | |  | | | oleoyl-ACP hydrolase | | | | | | |  |
| A_33_P3415350 | 0.0303 | | 2.05 | | | up | | | KCNB2 | | | potassium voltage-gated channel (KCNB2) | | | | | | |  |
| A_33_P3251342 | 0.0041 | | 2.05 | | | up | | | CYP3A4 | | | cytochrome P450, family 3, subfamily A, polypeptide 4 (CYP3A4) | | | | | | |  |
| A_33_P3315624 | 0.0043 | | 2.04 | | | up | | |  | | |  | | | | | | |  |
| A_33_P3405514 | 0.0282 | | 2.04 | | | up | | | MAGEA5 | | | melanoma antigen family A, 5 (MAGEA5) | | | | | | |  |
| A_24_P231026 | 0.0104 | | 2.04 | | | up | | | SCN8A | | | sodium channel, voltage gated, type VIII, alpha subunit (SCN8A) | | | | | | |  |
| A_33_P3211376 | 0.0395 | | 2.03 | | | up | | |  | | | GB | | | | | | |  |
| A_33_P3366296 | 0.0441 | | 2.01 | | | up | | | C14orf23 | | | chromosome 14 open reading frame 23 (C14orf23), variant 1 | | | | | | |  |
| A_23_P347029 | 0.0056 | | 2.01 | | | up | | | TAS2R50 | | | taste receptor, type 2, member 50 (TAS2R50) | | | | | | |  |
| A_33_P3285132 | 0.0172 | | 2.01 | | | up | | | ZNF599 | | | zinc finger protein 599 (ZNF599) | | | | | | |  |
| A_23_P21376 | 0.0173 | | 2.00 | | | up | | | MAGI2 | | | membrane guanylate kinase (MAGI2) | | | |  | | | |
| A_33_P3390122 | 0.0082 | | 2.00 | | | up | | | GIPC2 | | | GIPC PDZ domain containing family, member 2 (GIPC2) | | | |  | | | |
| **ProbeName** | **p-value*** | | **Fold change†** | | | **Regulation** | | | **GeneSymbol** | | | **Description** | | | | |  | | |
| A_33_P3296240 | 0.0127 | | 3.43 | | | down | | | CNTLN | | | centlein, centrosomal protein (CNTLN), transcript variant 1 | | | |  | | | |
| A_23_P133902 | 0.0046 | | 3.15 | | | down | | | PSORS1C1 | | | psoriasis susceptibility 1 candidate 1 (PSORS1C1) | | | |  | | | |
| **A_23_P157920** | **0.0359** | | **3.04** | | | **down** | | | **IFNA16** | | | **interferon, alpha 16 (IFNA16)** | | | |  | | | |
| A_23_P386320 | 0.0101 | | 2.11 | | | down | | | MFI2 | | | antigen p97 (melanoma associated) | | | | | | |  |
| A_24_P42446 | 0.0310 | | 2.06 | | | down | | | PURG | | | purine-rich element binding protein G (PURG) | | | | | | |  |
| A_24_P235338 | 0.0214 | | 2.04 | | | down | | | TRPA1 | | | transient receptor potential cation channel, subfamily A (TRPA1) | | | | | |  | |
|  |  | |  | | |  | | |  | | |  | | | | | | |  |
| *p-value T Test paired p-value P <= 0.05 | | | | | | | | †[Core] vs [βGal] ( Fold change >= 2.0 ) | | | | | | | | | |  | |
|  | |  | | |  | | |  | | |  | | |  | | | |  | |
